# The PTTG1/VASP axis promotes oral squamous cell carcinoma metastasis by modulating focal adhesion and actin filaments

**DOI:** 10.1002/1878-0261.13779

**Published:** 2025-01-10

**Authors:** Suyeon Park, Sang Shin Lee, Shihyun Kim, Yeonjun Lee, Gyeonwon Park, Jung Oh Kim, Jongho Choi

**Affiliations:** ^1^ Department of Oral Pathology, College of Dentistry Gangneung‐Wonju National University Korea; ^2^ Research Institute of Oral Science, College of Dentistry Gangneung‐Wonju National University Korea; ^3^ Data Science Center GENINUS Inc. Seoul Korea

**Keywords:** actin cytoskeleton, focal adhesion, oral squamous cell carcinoma, pituitary tumor‐transforming gene 1, vasodilator‐stimulated phosphoprotein

## Abstract

The dynamics of focal adhesions (FAs) are essential physiological processes involved in cell spreading, metastasis, and regulation of the actin cytoskeleton. FAs are complex structures comprising proteins, such as paxillin and zyxin, which interact with extracellular membranes and influence cell motility and morphology. Although related studies have been reported in various cancers, the function and molecular mechanisms of oral squamous cell carcinoma (OSCC) remain unknown. We investigated the coordination between the actin cytoskeleton and FA proteins, specifically introducing pituitary tumor‐transforming gene 1 (*PTTG1*; also known as PTTG1 regulator of sister chromatid separation, securin) into OSCC. Furthermore, we explored the co‐localization of several FAs and PTTG1 through small interfering RNA (siRNA) control or siRNA‐vasodilator‐stimulated phosphoprotein (*VASP*) and ‐*PTTG1*, examining the mechanisms mediated by the induced changes in OSCC both *in vitro* and *in vivo*. The knockdown of *VASP* and *PTTG1* regulates the dynamic actin cytoskeleton, restricting cell protrusion and motility from the front to the rear of OSCC cells. Our findings may provide new insights into how cells interact with each other on the surface of FAs in OSCC, influencing metastatic properties.

## Graphical abstract

VASP was found to be overexpressed in metastatic oral squamous cell carcinoma (OSCC) tissues. Notably, PTTG1‐ and VASP‐deficient OSCC cells demonstrated suppressed metastatic properties by disrupting the interaction between the cytoskeleton and focal adhesion (FAs) in the filopodia region. These findings highlight the potential for targeting these pathways in the treatment of OSCC metastasis.

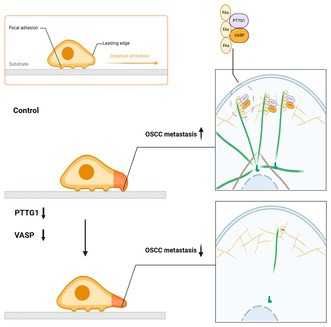

**Abbreviations**
BSA: bovine serum albumin
CNTL: control
DAPI: 4′,6‐diamidino‐2‐phenylindole
EVL: Enah/Vasp‐like
FA: focal adhesion
GAPDH: glyceraldehyde 3‐phosphate dehydrogenase
H&E: hematoxylin and eosin
IF: immunofluorescence
IHC: immunohistochemistry
Mena: mammalian‐enabled
OSCC: oral squamous cell carcinoma
PBS: phosphate‐buffered saline
PLA: Duolink Proximity Ligation Assay
PTTG1: pituitary tumor‐transforming gene 1
PXL: paxillin
RIAM: Rap1‐GTP‐interacting adapter molecule
siRNA: small interfering RNA
VASP: vasodilator‐stimulated phosphoprotein
VCL: vinculin
ZYX: zyxin

## 1 Introduction

Oral squamous cell carcinoma (OSCC) is the most common subtype of head and neck cell carcinoma, affecting the oral cavity, pharynx, and larynx, and accounts for over 90% of all malignant neoplasms of the oral cavity. In 2022, an estimated 390 000 (2.0%) new cases and 190 000 (1.9%) cancer‐related deaths were reported worldwide, based on GLOBOCAN 2022 [1]. Although the proportion is lower than that of other cancers such as lung, colorectal, prostate, and stomach cancers, it is important to note that it has been increasing since 2018. In addition, the prognosis of OSCC and the 5‐year‐survival rate of approximately 50% remain poor because of the potential for lymph node metastasis and rapid growth in the early stage [2, 3, 4]. Currently, OSCC patients undergo surgery as the principal treatment method for non‐metastatic OSCC, whereas patients with recurrent or metastatic OSCC are treated with radiation therapy or chemoradiation [5]. Nevertheless, these treatment methods cause aggressive side effects that lead to nonspecific cell death in patients, requiring targeted therapies with the advantages of high selectivity, therapeutic index, and low toxicity as novel OSCC treatment methods based on the understanding of metastatic characteristics.

Cell migration necessitates the orchestration of multiple processes, including substrate sensing, continuous and dynamic remodeling of the cell matrix, cell adhesion, and formation of protruding structures [6, 7, 8]. Specifically, regulated actin polymerization at the leading edge of the protrusive structure results in the development of broad, sheet‐like lamellipodia and thin, finger‐like filopodia [9, 10], which serve as sensory extensions that explore the cellular environment for guidance cues and function as adhesive structures, facilitating initial substrate attachment and signal transduction to govern the establishment and maturation of stable focal adhesions (FAs) [11, 12]. Ena/vasodilator‐stimulated phosphoprotein (Ena/VASP) proteins, which include three members in mammals (*Mena*), VASP, and Ena‐VASP‐like protein (*EVL*) [13], play crucial roles in regulating the cytoskeleton and dynamic actin filaments by localizing to active actin filaments in FAs, filopodia, and lamellipodia [14, 15, 16]. In hepatocellular carcinoma cells, *in vitro* studies have shown that Mena overexpression promotes tumor growth and migration through epithelial–mesenchymal transition (EMT) [17], which is associated with tumor cell motility, intravasation, and metastasis *in vivo* [18]. *VASP* is composed of three functional regions: an EVH1 domain at the N terminus, a proline‐rich (PRO) domain in the middle, and an EVH2 domain at the C terminus, which regulate actin filament dynamics [19]. Recent studies have reported a close relationship between *VASP* expression and tumor progression [20]. In hepatocellular carcinoma (HCC), *VASP* expression has been shown to promote metastasis [21]. Additionally, *VASP* overexpression has been linked to increased invasion ability in MDA‐MB‐231 cells, lymph node metastasis in breast cancer (BC) patients, and enhanced BC cell migration via interaction with integrin α3, a cell adhesion receptor [22, 23]. However, knowledge regarding Ena/VASP proteins and the underlying molecular mechanisms involved in OSCC is limited.

*PTTG1* was initially identified in rat pituitary tumor cells and has been shown to functionally induce the transformation of mouse fibroblasts into malignant tumors [24]. In human cells, *hPTTG1* also functions as a cell cycle regulator, participates in sister chromatid separation [25], and plays a significant role in regulating cancer cell transformation and tumor grade [26]. In particular, *PTTG1* is considered a signature gene that can predict and diagnose metastasis in various types of cancer [27], including those involving the EMT, and its overexpression is often associated with increased invasion and higher tumor grades, particularly in relation to the surrounding lymph nodes [28, 29]. In OSCC, *PTTG1* expression is associated with the development of OSCC in precancerous lesions [30], which is linked to lymph node metastasis and the tumor‐node‐metastasis (TNM) process, potentially through the EMT mechanism [31], research on the direct relationship and molecular mechanisms of OSCC metastasis involving *PTTG1*. In the present study, we investigated the effects of *PTTG1* on cell growth and motility, and its relationship with FA‐associated proteins and actin filaments in OSCC.

Here, we report for the first time the upregulation of *VASP* expression in patients with metastatic OSCC, highlighting its association with focal adhesion and actin filament dynamics. Additionally, our findings revealed the involvement of the *PTTG1*/*VASP* signaling pathway in regulating the metastatic properties of OSCC. These observations underscore the need for a comprehensive investigation of the functional and pathological roles of *VASP*/*PTTG1* in OSCC.

## 2 Materials and methods

### 2.1 TCGA database analysis

Gene expression data were downloaded from The Cancer Genome Atlas (TCGA) database (https://portal.gdc.cancer.gov). The data set contained total of 361 OSCC samples, including 32 adjacent tumor samples, which were visualized using r software (version 4.2.1; R Core Team, Vienna, Austria). The FPKM data were paired with 32 normal and tumor samples from patients with OSCC and transformed into transcripts per million (TPM) values following log_2_ (+1) normalization. To evaluate the tools generating continuous scores, receiver operating characteristic (ROC) analysis was performed for *PTTG1* and *VASP*. From each ROC curve, we determined the optimal decision threshold as the threshold with minimal difference between sensitivity and specificity [32, 33]. Differentially expressed gene (DEGs) analysis was performed on the count matrix of the samples using the r package, and deseq2 (http://www.bioconductor.org) was used to adjust *P*‐values to minimize false positives using the Benjamini and Hochberg method [34]. To visualize the correlation graph of FA‐related genes, Pearson correlation coefficients were used in r software as a heatmap of the correlation coefficient matrix. The screening conditions for the DEGs were log_2_ (fold change) > |1| and *P*‐values < 0.05 [35].

### 2.2 Patient tissues samples

Human OSCC tissue samples were obtained from 26 patients; non‐metastatic samples (*n* = 13), and metastatic samples (*n* = 13) at the Gangneung‐Wonju National University Dental Hospital, a member of the Korea Biobank Network (December 2020 to February 2023). Written informed consent was obtained from all patients, and the study was approved by the Research Ethics Review Committee of Gangneung‐Wonju National University (IRB No. GWNUIRB‐2020‐26‐1). The study included participants who underwent surgery and were histopathological diagnosis an aged from 50 to 90 years with OSCC. All study methodologies were performed according to the standards set by the Declaration of Helsinki.

### 2.3 Cell culture and transfection

The human OSCC cell line HSC‐2 (RRID: CVCL_1287), HSC‐3 (RRID: CVCL_1288) and HSC‐4 (RRID: CVCL_1289) were acquired from the Japanese Collection of Research Bioresources Cell Bank (Ibaraki, Osaka, Japan). SCC‐9 (RRID: CVCL_1685) cell line was obtained from the American Type Culture Collection (ATCC, Manassas, VA, USA), and YD‐10B (RRID: CVCL_8929) cell line was provided by Yonsei University College of Dentistry (Seoul, Korea). OSCC cell lines were cultured and maintained in Dulbecco's modified Eagle's medium (DMEM; Invitrogen, Carlsbad, CA, USA) supplemented with 10% fetal bovine serum (FBS; Gibco, Waltham, MA, USA) and 1% penicillin/streptomycin (P/S; Gibco). All cell lines were incubated in a humidified chamber at 37 °C with 5% CO_2_ and used for experiments after reaching full confluence. The cells were transfected and incubated with 50 nm of small interfering RNA (siRNA) targeting *VASP* (5′‐CGG CCA AUU CCU UUC GCG U‐3′) and *PTTG1* (5′‐AGC ACC AGA UUG CGC ACC U‐3′) (Bioneer, Daejeon, Korea) or, as a control, with non‐targeting siRNA were synthesized from Bioneer Co. for 24 h using Lipofectamine® 2000 (Invitrogen). The cell lines used were regularly tested for Mycoplasma contamination using PCR, and no positive Mycoplasma infection was detected (ATCC). The cell lines were authenticated through genetic fingerprinting and were used for no more than 20 passages upon cryogenic thawing.

### 2.4 Quantitative real‐time polymerase chain reaction analysis (qRT‐PCR) analysis

To quantitatively evaluate *PTTG1*, *VASP* and paxillin (PXN) mRNA levels, total RNA was isolated from mouse lung tissues using the TRIzol reagent (Invitrogen). The equal concentration of cDNA was synthesized using AccuPower® RocketScript Cycle RT PreMix (Bioneer) consisting of reverse transcriptase, RNase inhibitor, and deoxyNucleoside triphosphate (dNTP), and followed by 50 °C for 60 min, 95 °C for 5 min, and hold at 4 °C. qRT‐PCR was performed on a CFX96 Real‐Time PCR Detection System (Bio‐Rad Laboratories, Hercules, CA, USA) using SYBR Green Master Mix (Bioneer). The cycling conditions were followed by 40 cycles of 95 °C for 15 s, 60 °C for 30 s, 95 °C for 15 s, 65 °C for 5 s, and 95 °C for 30 s. The relative gene expression levels were evaluated using the 2^−ΔΔCt^ method, where ΔCt = Ct (gene of interest) − Ct (reference gene), and ΔΔCt = ΔCt and the mRNA levels of the target genes were normalized to that of GAPDH. Primer sequences used in this study are listed in Table S1.

### 2.5 Western blot assay

Cells were cultured on 60 mm plates (SPL Life Sciences, Seoul, Korea) with siRNA or a non‐targeting siRNA control for a day incubation. After incubation, total protein was scraped into 1× Laemmli buffer (Bio‐Rad) containing protease inhibitor cocktail (Roche Diagnostics, Basel, Switzerland). 10–20 μL of protein was loaded in 8–15% SDS/PAGE and transferred to nitrocellulose membrane 0.45 μm (GenDEPOT, Katy, TX, USA) for overnight. After transfer, the membranes were blocked with 5% bovine serum albumin (BSA; Sigma‐Aldrich, St. Louis, MO, USA) in 0.01% phosphate‐buffered saline Tween‐20 (PBS‐T) for 1 h at room temperature (RT, 20–25 °C) and incubated with primary antibodies at 4 °C. The following primary antibodies were used: mouse anti‐*EVL* (#sc‐373793; Santa Cruz Biotechnology, Santa Cruz, CA, USA), mouse anti‐*Mena* (#sc‐135988; Santa Cruz), mouse anti‐*VASP* (#sc‐46668; Santa Cruz), mouse anti‐*PTTG1* (#sc‐56207; Santa Cruz) rabbit anti‐*paxillin* (#50195; Cell Signaling Technology, Danvers, MA, USA), rabbit anti‐*zyxin* (#3553; Cell Signaling), rabbit anti‐*vinculin* (#13901; Cell Signaling) were used at 1/1000 dilution. Rabbit anti‐*GAPDH* (#LF‐PA0018; AB Frontier, Seoul, Korea) was used as an internal control. After washing with PBS‐T, the membranes were incubated with secondary antibodies with horseradish peroxidase (HRP)‐conjugated goat anti‐rabbit or mouse IgG (#7074 or #7076; Cell Signaling) for 1 h at RT. Proteins were detected using a chemiluminescent reagent (Millipore, St. Louis, MO, USA) and visualized using the FUSION Solo S Imaging System (Vilber, Eberhardzell, Germany). Protein intensities were quantified using imagej (National Institutes of Health, Bethesda, MD, USA). All experiments were conducted at least in triplicates. The information on the antibodies used in this study is provided in Table S2.

### 2.6 Duolink proximity ligation assay (PLA)

To investigate protein–protein interactions in OSCC cells after siRNA treatment, the PLA using *in situ* Duolink Red Kit (cat. #DUO92101; Sigma‐Aldrich), according to the manufacturer's protocol. Briefly, OSCC cells were seeded onto 30 mm with coverslips at a density of 1 × 10^4^ cells until they reached 40–60% confluence. After treated with siRNA or mock control, cells were fixed with 4% paraformaldehyde (PFA) for 15 min, and permeabilized with 0.5% Triton X‐100 for 15 min at RT and blocked with blocking solution for 1 h at 37 °C humidified incubator. The coverslips were blocked with the blocking solution and then incubated with mouse anti‐*VASP* (#sc‐46668; Santa Cruz) and rabbit anti‐*paxillin* (#50195; Cell Signaling), mouse anti‐*VASP* and rabbit anti‐*zyxin* (#3553; Cell Signaling), mouse anti‐*VASP* and rabbit anti‐*PTTG1* (#GTX111938; GeneTex, Inc., Irvine, CA, USA) or mouse anti‐*PTTG1* (#sc‐56207; Santa Cruz) and rabbit anti‐*paxillin*, in antibodies dilution buffer at 37 °C for 1 h. The coverslips were then washed with 1× wash buffer for 5 min and incubated with PLA probes 37 °C for 1 h. After a brief rinse with 1× wash buffer, the coverslips were incubated with ligation buffer at 37 °C for 30 min and amplified with anti‐rabbit and anti‐mouse PLA probe in dark humidified condition, and fixation with DAPI for 15 min at RT. Images were obtained using a confocal microscope (Leica Microsystems, Wetzlar, Germany) with a 63× objective oil lens equipped with Z‐stacks (sequential scan; a step size of 0.25 μm steps; 10 images). PLA signals with more than 5 pixels were captured, and the entire signal for each cell (protein–protein interactions) was counted using imagej Software.

### 2.7 Cell migration and invasion assay

To analyze the metastatic potential of OSCC cells, migration and invasion assay were conducted using Transwell chamber (8 μm pore size; Corning, Inc., Steuben County, NY, USA) according to the manufacturer's instructions. For migration assay, 8 × 10^3^ HSC‐2 and SCC‐9 cells were treated with 50 nm of siR‐*PTTG1* or ‐*VASP* and cultured in 300 μL of serum‐free medium for 24 h. In the bottom chamber, 700 μL of culture medium supplemented with 10% FBS were added to chemoattractant. Migrated cells were fixed with 100% methanol and stained with Mayer's hematoxylin staining reagent (cat. #3309; DAKO, Santa Clara, CA, USA), and incubated for 20 min at RT. Unstained cells were eliminated using a cotton swab and stained cells were observed under a light microscope (BX53; Olympus, Tokyo, Japan). For the invasion assay, the upper chamber was precoated with Matrigel (BD Biosciences, San Jose, CA, USA) before cell seeding, and the subsequent procedure was performed using a migration assay. The stained cells were captured in eight randomly selected fields using a 20× magnification lens, and quantification was performed using the imagej software.

### 2.8 Immunofluorescence (IF) labeling and confocal microscopy

5 × 10^4^ OSCC cells were plated and cultured with non‐targeting siRNA or siR‐*PTTG1* and ‐*VASP* onto 30 mm culture dishes (SPL) with 12 mm Φ cover slips (Marienfeld‐Superior, Lauda‐Konigshofen, Germany). Cultured cells were fixed with 4% PFA for 15 min, permeabilized with 0.5% Triton X‐100 for 15 min, and washed with PBS at RT. Mouse anti‐*EVL* (#sc‐373793; Santa Cruz), mouse anti‐*Mena* (#sc‐135988; Santa Cruz), mouse anti‐*VASP* (#sc‐46668; Santa Cruz), rabbit anti‐*paxillin* (#50195; Cell Signaling), rabbit anti‐*zyxin* (#3553; Cell Signaling), rabbit anti‐*vinculin* (#13901; Cell Signaling), rabbit anti‐*PTTG1* (#GTX111938; GeneTex), rabbit anti‐*β‐tubulin* (#2128; Cell Signaling), rabbit anti‐*RIAM* (#14300; Novus, Biological, LLC, Centennial, CO, USA) and rabbit anti‐*profilin‐1* (#3237; Cell Signaling) were used at 1/100 diluted in PBS and used as primary antibodies for 1 h at RT. After washed with PBS, the coverslips were incubated with Alexa Fluor® 488 or Alexa Fluor® 568 (A32723 and A‐11011; Invitrogen) were used as secondary antibodies for 1 h at RT. Alexa Fluor® 647 Phalloidin (#8940; Cell Signaling) was used for actin cytoskeleton at 1/50 diluted in PBS for 15 min at RT. The coverslips were washed and mounted with mounting medium containing DAPI (cat. #ab104139; Abcam, Waltham, MA, USA) for 15 min at RT. Images were acquired using a laser confocal microscope (Leica) at an optical resolution of 512 × 512 pixels with a 63× objective oil lens (above the z‐stack acquisition). The correlation between signals was measured using the BIOP plugin in imagej software. All experiments were performed at least in triplicate.

### 2.9 *In vivo* tumor metastatic mice model

Male BALB/c nude mice (8 weeks old; Orient Bio, Inc., Gyeonggi, Korea) were randomly assigned to each group (*n* = 5). All mice were housed in a specific pathogen‐free class laboratory animal room, which was maintained at 22 ± 2 °C on a regular light–dark cycle. All animals were provided with adequate food and water, and the study was approved by the Animal Ethics Committee of Gangneung‐Wonju National University, Korea (IRB No. GWNUIR2B‐2020‐26‐1).

Oral squamous cell carcinoma cells were collected separately in the logarithmic phase, labeled with Vybrant™ DID Cell‐Labeling Solution (Thermo Fisher Scientific, Waltham, MA, USA), washed with PBS 3 times and added to serum‐free media. The cells were counted and cell concentration was adjusted 1 × 10^6^ cells in 100 μL PBS. Inside the tail vein of nude mice, the cells were inoculated a 1 mL Insulin Syringe (BD Ultra‐Fine; Dickinson and Company, Franklin Lakes, NJ, USA). The mice were sacrificed at approximately 5 weeks of age, and lymph nodes were collected for human analysis, H&E (hematoxylin and eosin) staining, and production of paraffinized sections. To monitor tumor growth and metastasis within isolated lungs of nude mice, bioluminescence images were acquired for a total exposure time of 1 min using fusion image software (Vilber). The optical signal was calculated as the number of photons emitted per second, and captured images were observed and the diameter of the tumor was measured using cellsens standard software (Olympus).

### 2.10 Human‐specific Alu real‐time PCR primer

Human Alu sequences obtained by qRT‐PCR were used to evaluate the human DNA from OSCC cells isolated from mouse lung tissues. The concentration of cDNA was synthesized using AccuPower® RocketScript Cycle RT PreMix (Bioneer) and the conditions were described as above. Quantification of the Alu sequences was performed on a Real‐Time PCR Detection System (Bio‐Rad) using the SYBR Green Master Mix (Bioneer). The PCR conditions were followed by 35 cycles of 95 °C for 15 s, 60 °C for 60 s, 65 °C for 1 s, and 94 °C for 30 s. hAlu sequences were normalized to that of GAPDH. All experiments were performed in triplicate.

### 2.11 H&E staining

To observe the metastatic OSCC into lung tissues in mice model, the lung tissues were sliced with thickness 4 μm, and baked at 65 °C for 2 h. Sections were deparaffinized in xylene and gradually hydrated in a graded series of ethanol. The slides were stained with a hematoxylin solution (BBC Biochemical, Mount Vernon, WA, USA) diluted in distilled water (ddH_2_O) for 10 min, washed in ddH_2_O until the nuclei became blue, and counterstained with 1% eosin Y solution (Muto, Pure Chemicals Co., Tokyo, Japan) for 30 s. For dehydration, sections were serially diluted with ethanol and subsequently cleared in xylene. The slides were mounted with Canada balsam (Sigma‐Aldrich) and Images were observed with a microscope (Olympus) with a 20× objective, and at least five randomly selected fields were acquired.

### 2.12 Immunohistochemistry

All tissue samples were obtained on 4 μm paraffin section on 1.0 mm coated microscope slides (Muto Pure Chemicals Co., Ltd., Bunkyo, Tokyo, Japan). The sections were deparaffinized and rehydrated using an ethanol series. Antigen retrieval was performed in a microwave and nonspecific binding was blocked with 5% BSA at RT for 30 min. For incubation with primary mouse anti‐*VASP* (#sc‐46668; Santa Cruz) were applied overnight at 4 °C. The slides were rinsed with PBS‐T and incubated with secondary antibodies in 5% BSA at RT for 1 h. Staining was developed with 3,3′‐Diaminobenzidine (DAB) followed by hematoxylin (DAKO) counterstaining. After dehydration with serial dilutions of ethanol and xylene, the sections were mounted with a xylene mixture containing Canada balsam (Sigma‐Aldrich). The sections were evaluated and images were obtained using a microscope (Olympus) with a 20× objective. All experiments were conducted in triplicates.

### 2.13 Statistical analysis

All assays were repeated at least triplicate, and the data are presented as mean ± SEM. The significance of differences between the mock control and experimental groups was determined using an unpaired Student's *t*‐test. Statistical analyses were performed using spss (version 26.0; SPSS Inc., Chicago, IL, USA) and graphpad prism 9 (GraphPad Software, San Diego, CA, USA).

## 3 Results

### 3.1 *VASP* is associated with FAs and actin filament in OSCC cells

**Fig. 1:**
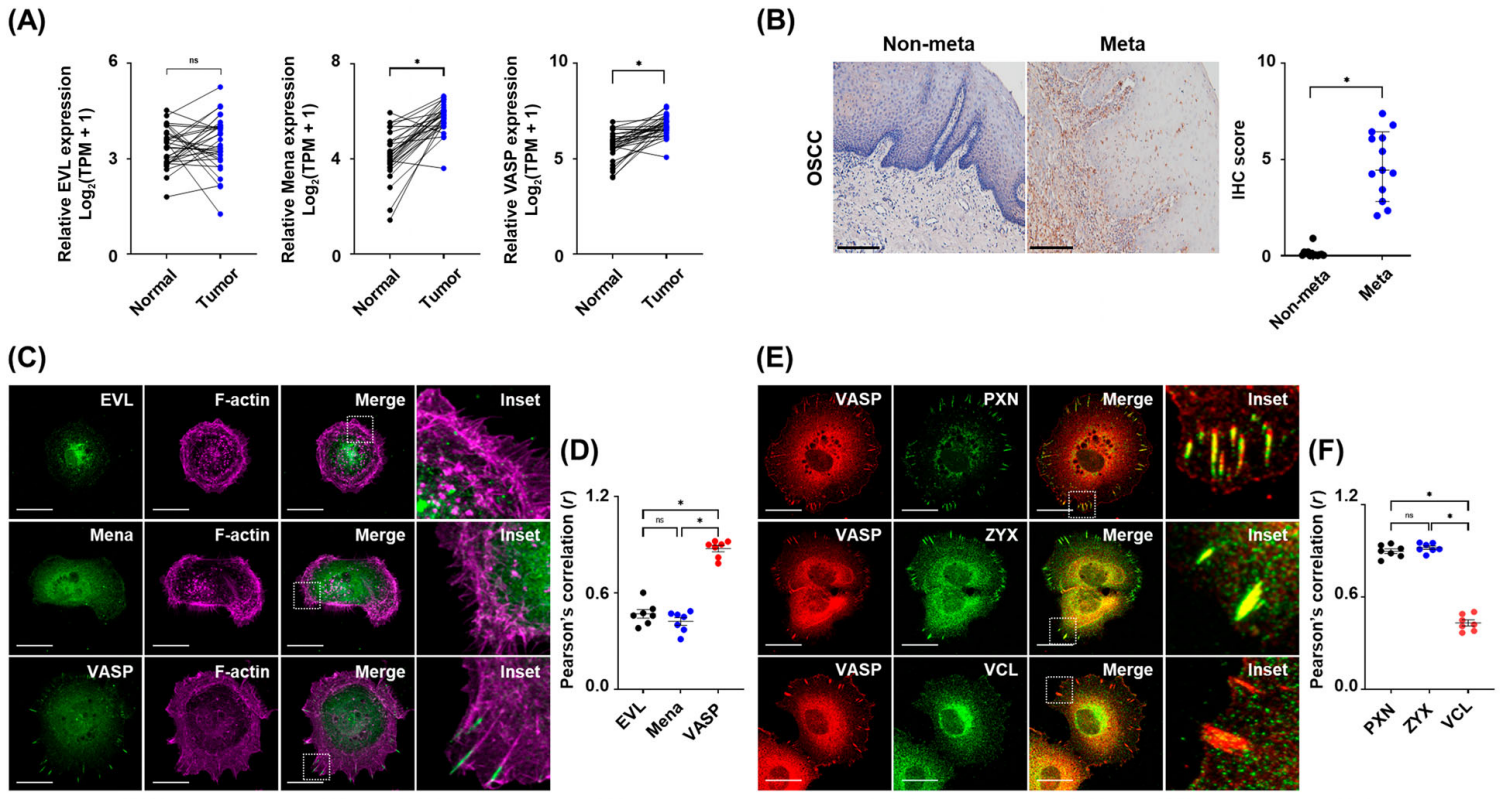
*VASP* expression correlates with the progression and metastasis of OSCC. (A) Expression level of *EVL*, *Mena* and *VASP* family between normal (*n* = 32) and OSCC samples (*n* = 32) evaluated in the TCGA cohort, respectively. ns, not significant; *P < 0.05. (B) Representative IHC images (left) and quantification (right) of *VASP* expression in non‐metastatic tissues (*n* = 13) and OSCC metastatic tissues (*n* = 13). Scatter plot showing the IHC scores of *VASP* in normal and OSCC metastatic tissues. Magnification: 20× and scale bar: 100 μm. (C) Representative immunofluorescence images and (D) Pearson's correlation coefficient (*r*) for co‐localization showing *EVL* (green), *Mena* (green), *VASP* (green) and *F‐actin* (purple) in HSC‐2 cells (*n* = 7). (E) Representative immunofluorescence images and (F) Pearson's correlation coefficient (*r*) for co‐localization showing *PXN* (green), *ZYX* (green) and *VCL* (green) with *VASP* (red) in HSC‐2 cells (*n* = 7). For all immunofluorescence images, magnification: 63× and scale bar: 20 μm. Data are presented as the means ± SEM of three independent experiments and statistical significance was analyzed by Student's *t*‐test, *P < 0.05. Experiments were performed in triplicate for individual treatment condition. *EVL*, enah/vasp‐like; IHC, immunohistochemistry; *Mena*, mammalian‐enabled; Meta, metastatic tissue; Non‐meta, non‐metastatic tissue; OSCC, oral squamous cell carcinoma; PXN, *paxillin*; *VASP*, vasodilator‐stimulated phosphoprotein; VCL, *vinculin*; ZYX, *zyxin*.

We examined the expression patterns of Ena/VASP family proteins in OSCC using public datasets. TCGA data revealed that the expression of *Mena* and *VASP* was significantly elevated in OSCC patient tissues compared to that in normal tissues, but not in *EVL* (Fig. 1A). Furthermore, immunohistochemistry (IHC) showed that the expression of *VASP* was elevated in metastatic tissues compared to that in non‐metastatic tissues (Fig. 1B). To explore the expression of *VASP* in OSCC and its association with actin filaments, we examined the co‐localization of *VASP* and *F‐actin* in OSCC cell lines (SCC‐9, HSC‐2, HSC‐3, HSC‐4, and YD‐10B). *VASP* was expressed at the leading edge of OSCC cell lines, and strong co‐localization with *F‐actin* was detected within filopodia in the following order: HSC‐2, SCC‐9, YD‐10B, HSC‐4, and HSC‐3 cell lines (Fig. S1). Consequently, to verify localization in subsequent experiments, HSC‐2 cells were selected for visualization, whereas SCC‐9 cells were used for comparative analysis. We found that *VASP* co‐localized with *F‐actin* and within the filopodia of HSC‐2 cells, but not with *Mena* or *EVL* (Fig. 1C), and the coefficient was more strongly correlated in *F‐actin* linked with *VASP* (*r* = 0.88) than with *EVL* (*r* = 0.47) and *Mena* (*r* = 0.42) (Fig. 1D). *VASP* is closely associated with FA adapter proteins, such as *paxillin*, *zyxin*, and *vinculin*, which are believed to play a crucial role in actin polymerization. Our results showed that *VASP* co‐localized within the filopodia region with *paxillin* and *zyxin*, but not with *vinculin* in HSC‐2 cells (Fig. 1E,F). These data suggest that Ena/VASP is independently expressed in OSCC cells and that the expression of *VASP* is associated with FA adapter proteins and actin filaments.

### 3.2 Deficiency of *VASP* impairs correlation with FAs and inhibits the metastatic properties of OSCC cells

**Fig. 2:**
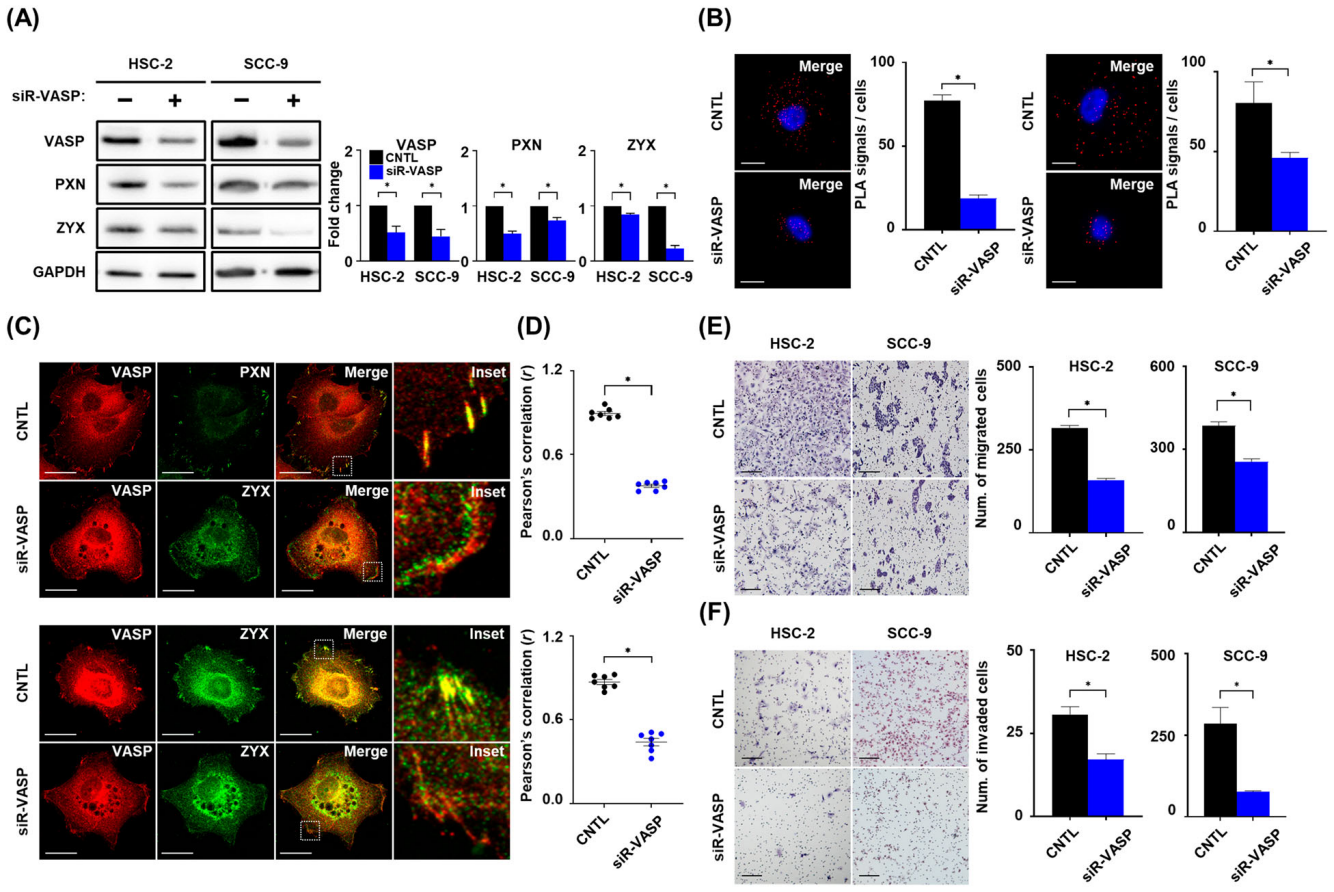
*VASP* knockdown suppresses the metastatic properties of OSCC cell lines by altering FAs. (A) Western blot analysis (left) and quantification (right) of *VASP* (46 kDa) and FA adaptor proteins (*PXN*, 68 kDa; *ZYX*, 74 kDa) in OSCC cell lines treated with siR‐*VASP* or control. *GAPDH* was used as loading control for normalization. (B) Proximity ligation assay (PLA) images and quantification showing co‐localization between *VASP* and *PXN* channels (left) or *VASP* and *ZYX* (right) treated with siR‐*VASP* or control of HSC‐2 cells. Magnification: 63× and scale bar: 20 μm. Red dots showing the proximity of *VASP* and *PXN* or *ZYX*. DAPI was used as a nuclear counterstain. (C) Representative immunofluorescence images and (D) Pearson's correlations (*r*) of *VASP* (red) with *PXN* (green, upper) and *ZYX* (green, lower) in HSC‐2 cells treated to siR‐*VASP* or control (*n* = 7). Magnification: 63× and scale bar: 20 μm. (E) Representative migration images (left) and quantification (right) and (F) representative invasion images (left) and quantification (right) of HSC‐2 and SCC‐9 cells transfected with siR‐*VASP* or control. Magnification 20× and scale bar: 50 μm. Data are presented as the means ± SEM of three independent experiments and statistical significance was analyzed using Student's *t*‐test, *P < 0.05. Experiments were performed in triplicate for individual treatment condition. CNTL, control; DAPI, 4′, 6‐diamidino‐2‐phenylindole; FAs, focal adhesions; *GAPDH*, glyceraldehyde 3‐phosphate dehydrogenase; OSCC, oral squamous cell carcinoma; PXN, *paxillin*; siR, small interfering RNA; VASP, vasodilator‐stimulated phosphoprotein; ZYX, *zyxin*.

To explore the role of *VASP* in FAs in OSCC cells, we used siRNA to knockdown *VASP* expression. Following siRNA‐*VASP* treatment, the expression of *paxillin*, *zyxin*, and *VASP* was significantly reduced in both HSC‐2 and SCC‐9 cell lines compared to that of the non‐targeting siRNA (control, Fig. 2A). We employed a single interaction‐sensitive PLA to detect the direct interactions between *VASP* and FAs. PLA is a powerful tool for visualizing the location of cellular proteins, providing a positive PLA signal that recognizes protein–protein interactions within 40 nm [36]. PLA analysis indicated that the number of PLA signals for both *VASP‐paxillin* (upper panel) and *VASP‐zyxin* (lower panel) was significantly reduced in HSC‐2 cells treated with siRNA‐*VASP* compared to that in the control group (Fig. 2B). A loss of co‐localization between *VASP* and *paxillin* (upper panel) or *VASP* and *zyxin* (lower panel) was observed in the filopodia of HSC‐2 cells treated with siRNA‐*VASP* (Fig. 2C). Moreover, the coefficients significantly decreased in HSC‐2 cells treated with siRNA‐*VASP* (upper panel: *VASP‐paxillin*: *r* = 0.38, lower panel: *VASP‐zyxin*: *r* = 0.42) compared to those in the control group (upper panel: *r* = 0.89; lower panel: *r* = 0.83) (Fig. 2D). Migration and invasion assays were performed to evaluate the metastatic properties of OSCC cells using *VASP*. The number of HSC‐2 and SCC‐9 cells that migrated from the upper chamber to the lower chamber was significantly reduced after siRNA‐*VASP* treatment (Fig. 2E). Similar to the migration assay, the number of invading cells was significantly lower in the siRNA‐*VASP* group than that in the control group (Fig. 2F). These findings suggest that the lack of *VASP* is disconnected from FAs, which inhibit the metastatic properties of OSCC cells.

### 3.3 *PTTG1* regulates the expression and localization of *VASP* in OSCC

**Fig. 3:**
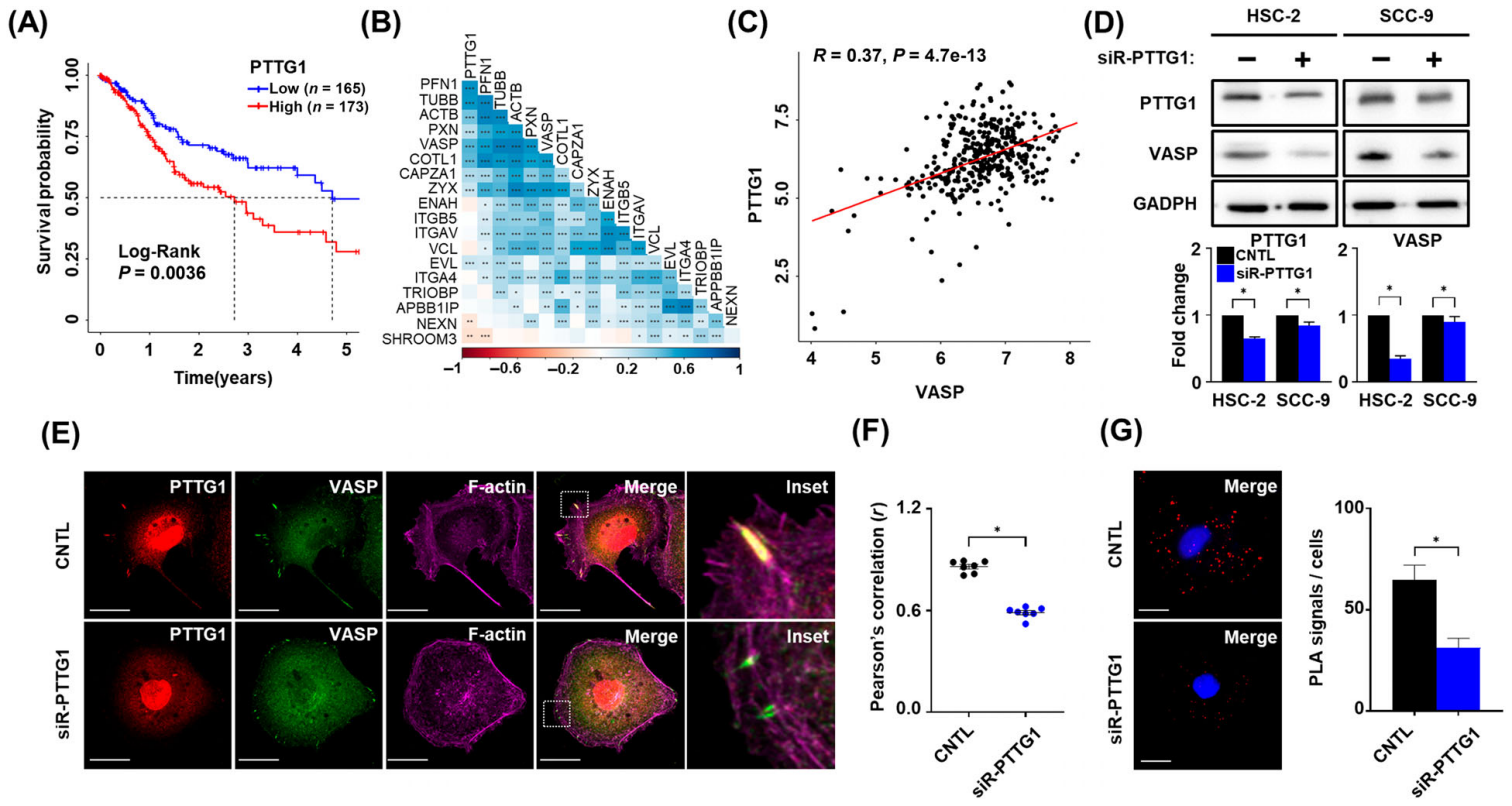
The expression of *PTTG1* correlates with the expression of *VASP* in OSCC. (A) Survival curves in the cohort of patients with OSCC, divided into two groups according to low (*n* = 165) and high (*n* = 173) PTTG1 expression level (*P* = 0.0036). (B) The correlation matrix plot of *PTTG1* related to the expressed genes (left, right), expression *P* value (center). The legends on the bottom that the colors used for visualization. (C) The positive correlation between *PTTG1* and *VASP* in OSCC (*R* = 0.37, *P* = 4.7e‐13). Red line indicates *R* value between *PTTG1* and *VASP*. (D) Western blot analysis (left) and quantification (right) of *PTTG1* (25 kDa) and *VASP* (46 kDa) in HSC‐2 and SCC‐9 treated with siR‐*PTTG1* or control. *GAPDH* was used as a loading control for normalization. (E) Representative immunofluorescence images and (F) Pearson's correlations (*r*) of *PTTG1* (red), *VASP* (green), and *F‐actin* (purple) in HSC‐2 cells treated with siR‐*PTTG1* or control (*n* = 7). Magnification: 63× and scale bar: 20 μm. (G) PLA images (left) and quantification (right) showing co‐localization between *PTTG1* and *VASP* channels treated with siR‐*PTTG1* or control of HSC‐2 cells. Magnification: 63× and scale bar: 20 μm. Red dots showing proximity of *PTTG1* and *VASP*. DAPI was used as a nuclear counterstain. Data are presented as the means ± SEM of three independent experiments and statistical significance was analyzed using Student's *t*‐test, *P < 0.05, **P < 0.01 and ***P < 0.001. Experiments were performed in triplicate for individual treatment condition. CNTL, control; DAPI, 4′, 6‐diamidino‐2‐phenylindole; *GAPDH*, glyceraldehyde 3‐phosphate dehydrogenase; OSCC, oral squamous cell carcinoma; PLA, proximity ligation assay; *PTTG1*, pituitary tumor‐transforming gene 1; siR, small interfering RNA; *VASP*, vasodilator‐stimulated phosphoprotein.

In our previous study, we demonstrated that *PTTG1* regulates the invasive ability of OSCC cells *in vitro* [36]. To further investigate the role of *PTTG1* in OSCC progression, we analyzed the TCGA data. Growth curve analysis indicated that the 5‐year‐survival rate of patients with high *PTTG1* expression was significantly lower than that of patients with low *PTTG1* expression (Fig. 3A). Correlation matrix plot results demonstrated that *PTTG1* expression was closely related to actin filament, *paxillin*, *zyxin*, and *VASP* expression in OSCC (Fig. 3B). Notably, a positive correlation was observed between *VASP* and *PTTG1* expression (Fig. 3C). To elucidate this *in vitro*, we suppressed the expression of *PTTG1* and examined its expression and co‐localization with *VASP*. Western blot data showed that the expression of *VASP* was significantly reduced in both HSC‐2 and SCC‐9 cell lines compared to that of the non‐targeting siRNA (control, Fig. 3D). In addition, IF data demonstrated that *PTTG1* co‐localized with *VASP* and *F‐actin* within the filopodia of HSC‐2 cells. However, after inhibition of *PTTG1* expression, the length of *VASP* within the filopodia was shortened, and the co‐localization of *PTTG1, VASP*, and *F‐actin* was significantly reduced in HSC‐2 cells (Fig. 3E,F). Furthermore, we observed the interaction between *PTTG1* and *VASP* using the PLA assay, and this interaction was interrupted in HSC‐2 cells treated with siRNA‐*PTTG1* compared to that in the control (Fig. 3G). These results suggest that *PTTG1* plays a regulatory role in the localization and expression of *VASP* in OSCC cells.

### 3.4 Deficiency of *PTTG1* regulates the FAs and suppresses the metastatic properties of OSCC *in vitro*

**Fig. 4:**
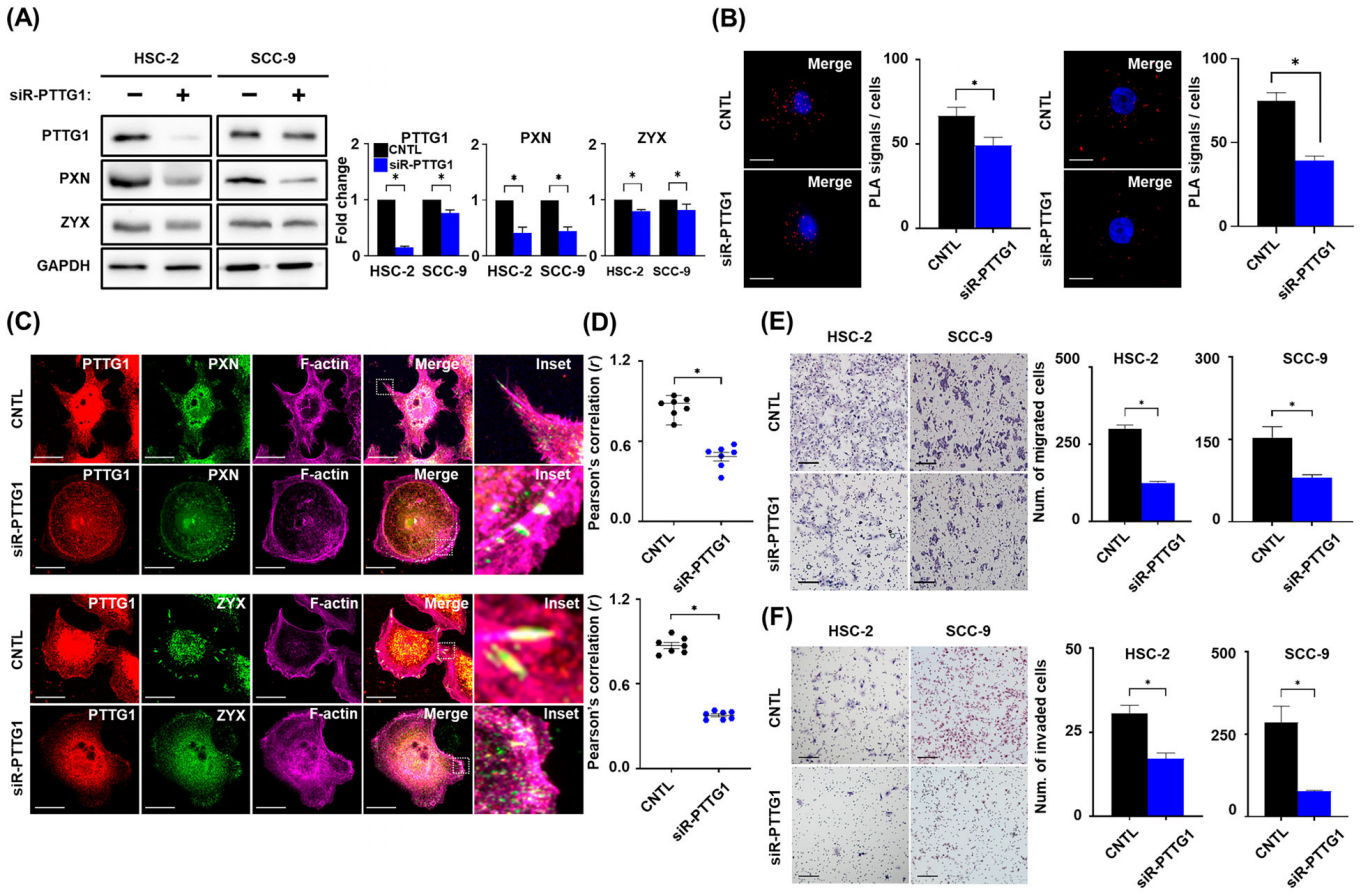
*PTTG1* knockdown suppresses the metastatic properties of OSCC cell lines by altering FAs and actin filaments. (A) Western blot analysis (left) and quantification (right) of PTTG1 (25 kDa), *PXN* (68 kDa) and *ZYX* (74 kDa) in HSC‐2 and SCC‐9 treated with siR‐*PTTG1* or control. *GAPDH* was used as a loading control for normalization. (B) PLA images and quantification showing co‐localization between *PTTG1* and *PXN* channels (left) or *VASP* and *ZYX* (right) treated with siR‐PTTG1 or control of HSC‐2 cells. Red dots showing the proximity of PTTG1 and *PXN*. DAPI used as a nuclear counterstain. Magnification: 63× and scale bar: 20 μm. (C) Representative immunofluorescence images of *PTTG1* (red) with *PXN* (green, upper), *ZYX* (green, lower), and *F‐actin* (purple) in HSC‐2 cells treated with siR‐*PTTG1* or control. Magnification: 63× and scale bar: 20 μm. (D) Pearson's correlations (*r*) of *PTTG1* with *PXN* (upper) and *ZYX* (lower) in HSC‐2 cells treated with siR‐*PTTG1* or control (*n* = 7). (E) Representative migration images (left) and quantification (right) and (F) representative invasion images (left) and quantification (right) of HSC‐2 and SCC‐9 cells transfected with siR‐*PTTG1* or control. Magnification 20× and scale bar: 50 μm. Data are presented as the means ± SEM of three independent experiments and statistical significance was analyzed using Student's *t*‐test, *P < 0.05. Experiments were performed in triplicate for individual treatment condition. CNTL, control; DAPI, 4′, 6‐diamidino‐2‐phenylindole; FAs, focal adhesions; *GAPDH*, glyceraldehyde 3‐phosphate dehydrogenase; PLA, proximity ligation assay; *PTTG1*, pituitary tumor‐transforming gene 1; *PXN*, paxillin; siR, small interfering RNA; *ZYX*, zyxin.

Our next challenge was to investigate whether the alteration in *VASP* resulting from *PTTG1* inhibition also affected FAs. The protein expression of FAs was decreased in HSC‐2 and SCC‐9 cell lines treated with siRNA‐*PTTG1* compared to that in the control group (Fig. 4A). In addition, the PLA assay showed that the number of positive PLA signals for both of *PTTG1‐paxillin* (left panel) and *VASP‐zyxin* (right panel) prominently decreased in HSC‐2 cells treated with siRNA‐*PTTG1* compared to the control (Fig. 4B). In control cells, *PTTG1*, FAs, and *F‐actin* were observed to co‐localize within the filopodia of HSC‐2 cells. However, in cells in which *PTTG1* was inhibited, the lengths of *paxillin* (upper panel) and *zyxin* (lower panel) were shortened, and *F‐actin* was limited to lamellipodia (Fig. 4C). After the inhibition of *PTTG1* expression, the correlation coefficients between *PTTG1*, FAs, and *F‐actin* were dramatically reduced (*r* = 0.86, *r* = 0.49) (Fig. 4D). Similar to the metastatic experiments conducted by inhibiting *VASP* in OSCC cell lines, the migration abilities of both HSC‐2 and SCC‐9 cells were significantly reduced following inhibition *PTTG1* (Fig. 4E). Additionally, the invasion ability decreased in both cell types (Fig. 4F). Alterations in FAs expression due to *PTTG1* may play a role in regulating the metastatic properties of OSCC cells.

### 3.5 Deficiency of *PTTG1* and *VASP* affects metastatic cell adhesion of OSCC *in vivo*

**Fig. 5:**
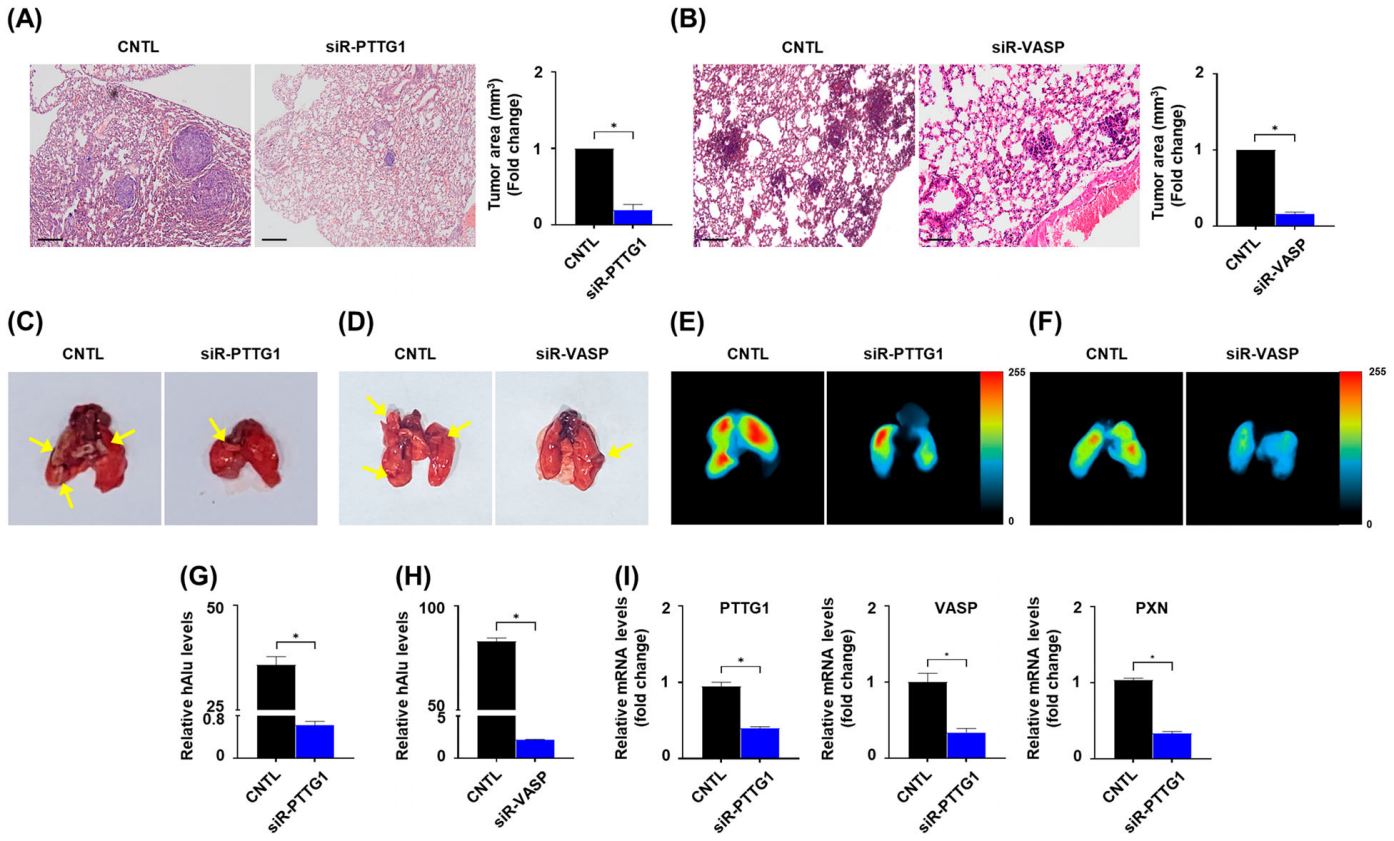
*PTTG1/VASP* knockdown suppresses the OSCC metastasis *in vivo*. (A) Representative H&E staining images (left) and quantification of tumor tissues (right) from nude mice after treatment with siR‐*PTTG1* or control in HSC‐2 cells. (B) Representative H&E staining images (left) and quantification of tumor tissues from nude mice after treatment with siR‐*VASP* or control in HSC‐2 cells. Magnification: 20× and scale bar: 100 μm. Representative images of the excised nude mouse lungs treated with (C) siR‐*PTTG1* (*n* = 5) or (D) ‐*VASP* (*n* = 5) compared with control in HSC‐2 cells; yellow arrows indicate metastasis lesions within nude mice. Representative biofluorescence images of nude mice lung treated to (E) siR‐*PTTG1* or (F) ‐*VASP* compared with control in HSC‐2 cells. The scale bar indicates the normalized fluorescent intensity. qRT‐PCR analysis showing *hAlu* in lung tissue after treatment with (G) siR‐*PTTG1* or (H) ‐*VASP* in HSC‐2 cells. (I) qRT‐PCR analysis showing *PTTG1*, *VASP* and PXN expression within nude mice tumor treated with siR‐*PTTG1* or control in HSC‐2 cells. Data are presented as the means ± SEM of three independent experiments and statistical significance was analyzed using Student's *t*‐test, *P < 0.05. Experiments were performed in triplicate for individual treatment condition. CNTL, control; H&E, hematoxylin and eosin; *PTTG1*, pituitary tumor‐transforming gene 1; siR, small interfering RNA; *VASP*, vasodilator‐stimulated phosphoprotein.

Lymph node metastasis is a common occurrence in OSCC patients [37], and recent studies have shown that cell adhesion plays a critical role in metastasis [38]. To validate the metastatic cell adhesion of OSCC *in vivo*, we administered tail vein injections of siRNA‐*PTTG1* or *VASP* and non‐targeting siRNA in nude mice. The lungs of nude mice were harvested and stained with hematoxylin and eosin (H&E) to identify metastatic nodules, which were significantly decreased by transfection with siRNA‐*PTTG1* group compared to control (Fig. 5A), and by transfection with siRNA‐*VASP* group compared control (Fig. 5B). *PTTG1* and *VASP* knockdown noticeably reduced the metastatic cell adhesion of tumor cells to the lungs after tail vein injection by treatment with siRNA‐*PTTG1* (Fig. 5C) or siRNA‐*VASP* (Fig. 5D), compared with the control. A bioluminescence imaging assay showed that metastatic nodules were significantly weakened after treatment with siRNA‐*PTTG1* (Fig. 5E) or siRNA‐*VASP* (Fig. 5F) compared to the control. In addition, the expression of *hAlu* was reduced following treatment with siRNA‐*PTTG1* (Fig. 5G) and siRNA‐*VASP* (Fig. 5H), compared to non‐targeting siRNA. Finally, the mRNA levels of *VASP* and *paxillin* was significantly decreased after transfection with siRNA‐*PTTG1* compared to the control (Fig. 5I). These mouse models indicate that the depletion of *PTTG1* and *VASP* represses metastatic tumor cell adhesion of OSCC *in vivo*.

## 4 Discussion

The metastasis of OSCC to lymph nodes poses a significant treat, frequently leading to cancer‐related mortality with limited treatment options. Therefore, it is essential to understand the complex processes of cell detachment, cell motility regulation, and invasion associated with metastasis [39]. Mechanistically, in motile cells, this process consists of three sequential steps that are dependent on FAs. Initially, protrusions are formed at the leading edge of the cell in response to guidance signals from the extracellular matrix (ECM). Subsequently, new FAs are formed at the leading edge of the cell and exhibit strong attachment to the ECM. Finally, FAs in the rear part of the cell are disassembled, facilitating forward movement of the entire cell body [40, 41]. However, the precise molecular mechanisms that link FAs to OSCC metastasis are poorly understood.

Focal adhesions are cellular components that connect the ECM to the cytoskeleton of cells, play a crucial role in cell migration by allowing cells to sense and respond to their environment, and interact organically with cytoskeletal components, such as actin filaments and microtubules (MTs), during cancer cell metastasis [42, 43]. Although several recent studies have shown that *VASP* plays a crucial role in cell mobility at the leading edge, where it engages with FAs such as *paxillin*, *vinculin*, and the integrin superfamily, contributing to the actin cytoskeleton, how *VASP* affects FAs is not well understood [44, 45]. To explore the interaction between *VASP* and specific FAs in OSCC, we selected OSCC cell lines that exhibited high metastatic potential and ease of observing filopodia and FAs. In our previous study, we demonstrated that the HSC‐2 cell line has aggressive metastatic potential compared to other OSCC cell lines [46]. Therefore, we deemed HSC‐2 cell lines as an ideal source for studying OSCC metastasis compared to other OSCC cell lines. Furthermore, in the present study, the formation of filopodia was most pronounced in the cell‐free edge region of HSC‐2 cells, accompanied by distinct expression of *VASP* and FAs, compared to other OSCC cell lines (Fig. 1E and Fig. S1). Consequently, the HSC‐2 cell line was selected for both *in vitro* and *in vivo* experiments to investigate the effect of *VASP* on the cytoskeleton and FAs, whereas the SCC‐9 cell line was used as a comparative cell line *in vitro*. Our results contradicted TCGA predictions, as we did not observe a direct association between *VASP* and MTs in OSCC cell lines. Instead, we found that the intracellular distribution of the two cytoskeletal components appeared distinct from each other, with MTs being widely distributed throughout the cell cytoplasm, while actin filaments were confined mainly to the cell‐free edge of OSCC cells, and their distribution in this region was directly correlated with the presence of *VASP* (Figs S1 and S2A). As demonstrated in previous studies, we attempted to determine whether the Rap‐1 GTP‐interacting adapter molecule (*RIAM*) and profilin, known as adapter proteins, were linked to *VASP* to establish a connection between the end of actin filaments and MTs [43, 47, 48, 49], but no direct evidence was found (Fig. S2B,C). This suggests that *VASP* interacts with the cell‐free edge of OSCC cells, highlighting its pivotal role as a modulator that interacts with actin filaments via co‐distribution.

At the cell‐free edge, the membrane containing lateral streams and bundles of actin filaments is lamellipodium, also known as microspikes, which are formed through the assembly of actin filaments [50]. As these microspikes transformed and protruded, filopodia were generated at the edges of the lamellipodium [51]. Although both structures are located parallel to actin filaments and share components, they are considered independent as the distribution of filopodia increases, even in the absence of a lamellipodium [52, 53]. Furthermore, there is insufficient research on the molecular mechanisms regulating these two structures in OSCC and their association with the metastatic potential of OSCC. We found that *EVL* and *Mena* were not distributed, and only *VASP* was observed in both structures. In cells lacking *VASP*, the lamellipodium form was still present, but the filopodia disappeared (Fig. 2B). While previous studies on other cancer types have highlighted the role of *VASP* interaction with FA adapter protein including vinculin, integrin superfamily and *RIAM* in promoting metastasis [23, 45, 54], our investigation did not reveal the co‐expression of *VASP* with these FA adapter proteins in the filopodia region of OSCC cell lines (Fig. 1E and Fig. S2). Notably, only *paxillin* and *zyxin* were detected together in the actin regulatory region, and their co‐expression was diminished in *VASP*‐deficient cell lines along with actin filaments, which suppressed the metastatic potential of OSCC *in vitro* and *in vivo*. Our results implied that *VASP* predominates over other proteins in OSCC cells. Specifically, *VASP* is distributed in filopodia and co‐localizes with FAs, suggesting that *VASP* distribution may contribute to the metastatic potential of OSCC.

*PTTG1* is a potent oncogene that drives malignant tumor progression via diverse mechanisms, prompting numerous investigations into the metastases associated with its overexpression [55, 56, 57, 58, 59]. The significance of *PTTG1* overexpression in tumor development, as emphasized in previous studies, was further underscored by our recent findings, with the present study elucidating the role of *PTTG1* in OSCC metastasis [36]. However, in the present study, we found that *PTTG1* expression enhanced the dynamic interaction between FAs and actin filaments in OSCC. Additionally, we demonstrated for the first time that silencing *PTTG1* not only decreased the distribution of *VASP* but also inhibited the metastatic potential of OSCC cells by disrupting the interactions between *PTTG1* and *VASP* and FAs in the filopodia region. Li et al. reported that the inhibition of *PTTG1* leads to actin filament depolymerization through cofilin and actin‐associated proteins and suppresses the invasion ability of lung cancer [60]. Although this finding supports our current findings, the relevance of *PTTG1* in *VASP* should be carefully considered. In contrast to the effect observed with *VASP*, *PTTG1* inhibition resulted in several differences in cell protrusions. Specially, *PTTG1* inhibition led to a decrease in both types of cell protrusions, including lamellipodia and filopodia (Fig. 3E). Second, while *PTTG1* inhibition demonstrated a suppressive effect on both *VASP* and FAs, a reduction, rather than complete disappearance, was observed (Figs 3E and 4C). Therefore, these issues should be thoroughly investigated in future studies to provide a more comprehensive understanding.

## 5 Conclusions

Our findings reveal that the expression and localization of *VASP* and *PTTG1* play critical roles in the metastasis of OSCC. We demonstrated that the interaction between *VASP* or *PTTG1* and focal adhesion adaptor proteins regulates the migration and actin polymerization. Both *PTTG1* and *VASP* deficiencies suppressed OSCC metastasis by constraining filopodia‐mediated focal adhesions (FAs) and disrupting actin filament dynamics. Thus, *VASP/PTTG1* appears to serve as an oncogene that is crucial for OSCC metastasis, suggesting that inhibiting *PTTG1/VASP* expression may represent a promising strategy for the treatment of metastatic OSCC. These novel insights enhance our understanding of the mechanisms underlying OSCC development and metastasis, potentially paving the way for new diagnostic and therapeutic strategies for OSCC metastasis.

## Conflict of interest

The authors declare no conflict of interest.

## Author contributions

SP and JC designed the study wrote the paper. SP and SK was responsible for formal analysis and investigation. YL, GP and JOK were responsible for data curation, validation and reviewing. SP and SK revised the manuscript. SSL and JC were responsible for funding acquisition, supervision and reviewing. All the authors have read and approved the final version of the manuscript.

### Peer review

The peer review history for this article is available at https://www.webofscience.com/api/gateway/wos/peer‐review/10.1002/1878‐0261.13779.

## Supporting information


**Fig. S1.** Confocal fluorescence images of OSCC cell lines stained with VASP (green) and F‐actin (purple).

**Fig. S2.** Confocal fluorescence images for genes involved in cell motility in HSC‐2.

**Table S1.** List of primer sequences used in the study.

**Table S2.** List of antibodies used in the study.

## Data Availability

The datasets used and analyzed in the present study are available from the corresponding author upon reasonable request.
