# Supplementary figures and images for: The PTTG1/VASP axis promotes oral squamous cell carcinoma metastasis by modulating focal adhesion and actin filaments
Source: Mol Oncol. 2025 Jan 10;19(5):1517–31. doi: 10.1002/1878-0261.13779 (PMC12077276; doi:10.1002/1878-0261.13779)

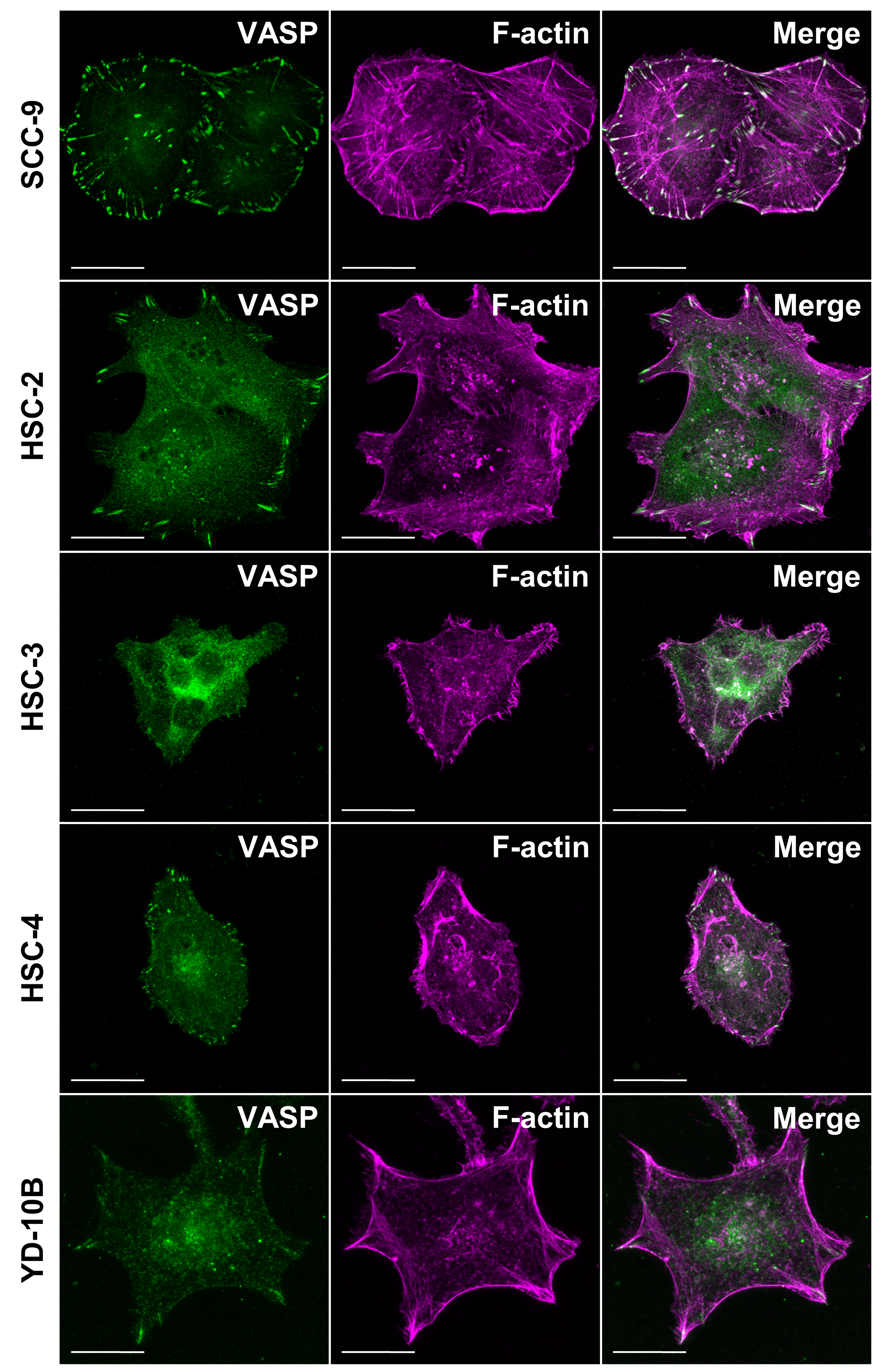

Supplement: Supplementary file 1 — Fig. S1. Confocal fluorescence images of OSCC cell lines stained with VASP (green) and F‐actin (purple). [file MOL2-19-1517-s001.tif]

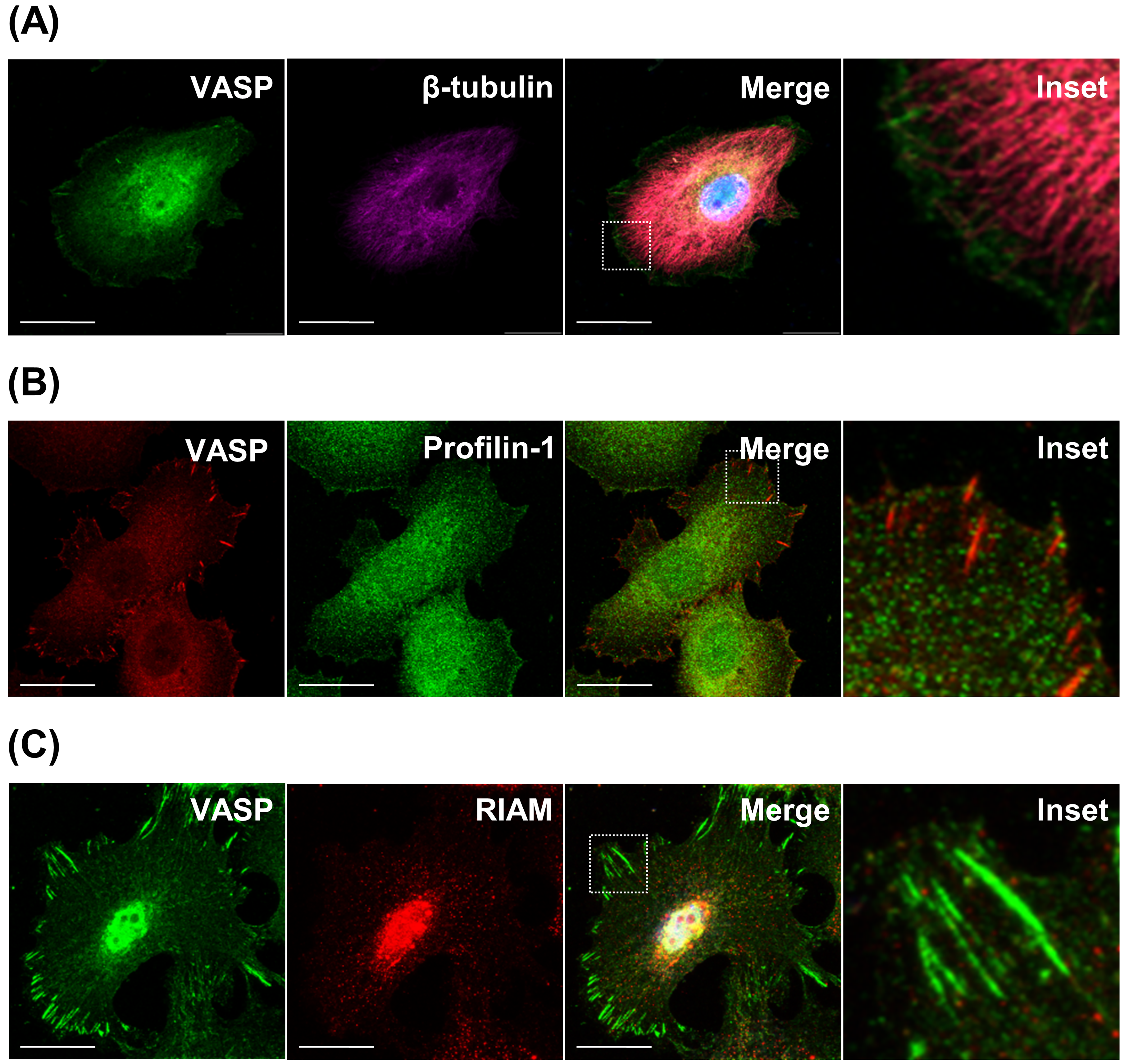

Supplement: Supplementary file 2 — Fig. S2. Confocal fluorescence images for genes involved in cell motility in HSC‐2. [file MOL2-19-1517-s003.tif]
