# Supplementary material for: The PTTG1/VASP axis promotes oral squamous cell carcinoma metastasis by modulating focal adhesion and actin filaments
Source: Mol Oncol. 2025 Jan 10;19(5):1517–31. doi: 10.1002/1878-0261.13779 (PMC12077276; doi:10.1002/1878-0261.13779)
Supplement: Supplementary file 3 — Table S1. List of primer sequences used in the study. [file MOL2-19-1517-s002.docx]

**Supplementary Table 1.** List of primer used in experiments.

| **Name** | **Gene ID** | **Sequence** |
| --- | --- | --- |
| *hPTTG1* |  | Forward: 5′- TGA CTC AGG CTG GAA GAT TTG -3′ |
|  |  | Reverse: 5′- GGT GGG AGA AGC AAA GGT ATA G -3′ |
| *hVASP* |  | Forward: 5′- GGA GCC AAA CTC AGG AAA GT -3′ |
|  |  | Reverse: 5′- CAT GGC GTT CAT CTC TTC CA -3′ |
| *hPaxillin* |  | Forward: 5′- GTT CCA AGT CCA GTA CCA AG -3′ |
|  |  | Reverse: 5′- GGA AGC CAC AGG AGT AAA C -3′ |
| *hZyxin* |  | Forward: 5′- CCT GGA TAA AGT GGT GAC AG -3′ |
|  |  | Reverse: 5′- TTG AGG GCT GAG ATA TAG -3′ |
| *hGAPDH* |  | Forward: 5′- CAA AGT TGT CAT GGA TGA CC -3′ |
|  |  | Reverse: 5′- CCA TGG AGA AGG CTG GGG -3′ |
| *hAlu* |  | Forward: 5′- CAT GGT GAA ACC CCG TCT CTA -3′ |
|  |  | Reverse: 5′- GCC TCA GCC TCC CGA GTA G -3′ |

h, human.
