# Supplementary material for: The PTTG1/VASP axis promotes oral squamous cell carcinoma metastasis by modulating focal adhesion and actin filaments
Source: Mol Oncol. 2025 Jan 10;19(5):1517–31. doi: 10.1002/1878-0261.13779 (PMC12077276; doi:10.1002/1878-0261.13779)
Supplement: Supplementary file 4 — Table S2. List of antibodies used in the study. [file MOL2-19-1517-s004.docx]

| **Primary antibody** | **Manufacturer** | **Catalog number** | **Class** | **Working dilution** | | |
| --- | --- | --- | --- | --- | --- | --- |
|  |  |  |  | **IHC** | **Western blot** | **IF** |
| *Anti-EVL* | Santa Cruz | sc-373739 | Monoclonal |  |  | 1:100 |
|  |  |  | mouse |  |  |  |
| *Anti-Mena* | Santa Cruz | sc-135988 | Monoclonal |  |  | 1:100 |
|  |  |  | mouse |  |  |  |
| *Anti-VASP* | Santa Cruz | sc-46668 | Monoclonal | 1:100 | 1:1,000 | 1:100 |
|  |  |  | mouse |  |  |  |
| *Anti-PTTG1* | Santa Cruz | sc-56207 | Monoclonal |  | 1:1,000 | 1:100 |
|  |  |  | mouse |  |  |  |
| *Anti-PTTG1* | Genetex | GTX111938 | Monoclonal  rabbit |  |  | 1:100 |
| *Anti-paxillin* | Cell signaling | #50195 | Monoclonal  rabbit |  | 1:1,000 | 1:100 |
| *Anti-zyxin* | Cell signaling | #3553 | Monoclonal  rabbit |  | 1:1,000 | 1:100 |
| *Anti-vinculin* | Cell signaling | #13901 | Monoclonal  Rabbit |  |  | 1:100 |
| *Anti-β-tubulin* | Cell signaling | #2128 | Monoclonal  rabbit |  |  | 1:100 |
| *Anti-RIAM* | Novus | #14300 | Monoclonal  rabbit |  |  | 1:100 |
| *Anti-profilin-1* | Cell signaling | #3237 | Monoclonal  rabbit |  |  | 1:100 |
| *Anti-GAPDH* | AB Frontier | LF-PA0018 | Monoclonal  rabbit |  | 1:3,000 |  |
| **Secondary antibody** | **Manufacturer** | **Catalog number** | **Class** | **Working dilution** | | |
|  |  |  |  | **IHC** | **Western blot** | **IF** |
| *Anti-rabbit IgG* | Cell signaling | #7074 | Mono/polyclonal |  | 1:5,000 |  |
| *Anti-mouse IgG* | Cell signaling | #7076 | Monoclonal | 1:100 | 1:5,000 |  |
| *Alexa Fluor 488* | Invitrogen | A32723 | Polyclonal |  |  | 1:100 |
| *Alexa Fluor 568* | Invitrogen | A11011 | Polyclonal |  |  | 1:100 |
| *Alexa Fluor 647 Phalloidin* | Cell signaling | #8940 |  |  |  | 1:100 |

**Supplementary Table 2.** List of antibodies used in the study.

IHC, Immunohistochemistry; IF, Immunofluorescence
